# Utilization of Ocular Ultrasound in the Evaluation of Posterior Reversible Encephalopathy Syndrome: A Case Report

**DOI:** 10.7759/cureus.4646

**Published:** 2019-05-11

**Authors:** David H Cisewski, Alfred J Astua

**Affiliations:** 1 Emergency Medicine, Icahn School of Medicine at Mount Sinai Hospital, New York, USA; 2 Pulmonary / Critical Care, Elmhurst Hospital Center, Mount Sinai School of Medicine, New York, USA

**Keywords:** ultrasound, intra-ocular pressure, posterior reversible encephalopathy, pres, neuro critical care, emergency

## Abstract

Posterior reversible encephalopathy syndrome (PRES) is an infrequently encountered cause of altered mental status and seizure activity in the emergency setting. Diagnosis is often delayed by extensive testing and failure to consider PRES in the differential. Though MRI remains the gold standard for diagnosis, ultrasound-guided measurement of intra-ocular pressure is a safe, effective alternative that can expedite the diagnosis. The treatment of PRES involves the rapid reversal of offending agents and aggressive blood pressure management. The prognosis of PRES is favorable and neurologic sequelae are uncommon. This clinical case highlights the importance of the emergency physicians’ consideration of this pathology and the utilization of ultrasound as a non-invasive means of assessing intra-ocular pressure.

## Introduction

Posterior reversible encephalopathy syndrome (PRES) is an uncommon but debilitating pathology seen in the emergency department (ED). The classic presentation of PRES includes seizures, altered mental status, and vision changes encompassing a broad differential. Due to the severity of alternative diagnoses, an expedited assessment for life-threatening mimickers such as subarachnoid hemorrhage, cerebral venous thrombosis, herpes simplex virus (HSV) encephalitis, alcohol withdrawal, or drug intoxication is essential. Though definitive diagnosis of PRES is made by MRI, the preceding workup may be extensive and invasive and an early evaluation of intra-ocular pressure using point-of-care ultrasound (POCUS) may accelerate diagnostic confirmation and reduce the need for further invasive testing. This article presents a case of an early diagnosis of PRES utilizing ultrasound guidance, as well as an overview of the diagnosis, treatment, and management of PRES in the emergency setting.

## Case presentation

A middle-aged female, 55 years old, with unknown past medical history (no acquaintances, no identification) presented to the emergency department by emergency medical services (EMS) with altered mental status in the setting of a witnessed seizure. Thirty minutes prior to arrival, bystanders noted the patient having full-body shaking while on the ground, which self-resolved after five minutes.

The patient's vital signs on presentation to the ED included a blood pressure of 162/107 mm Hg, heart rate of 83 beats per minute, respiratory rate of 17 breaths per minute (saturation 97% on room air) and a tympanic temperature of 97.5 F. On physical exam, the patient appeared disheveled, unkempt, fatigued, and with no obvious evidence of alcohol or drug intoxication. The patient was able to open her eyes and move all extremities, though she remained altered, lethargic, and neither spoke nor followed commands. Pupils were equal and reactive to light bilaterally. No skin rashes, lesions, or flushing were noted. A small hematoma was visualized on the posterior aspect of her head without active bleeding. Cranial nerve examination could not be conducted secondary to an inability to follow commands. No evidence of peripheral hyperreflexia was noted.

Initial lab results including basic metabolic panel, venous blood gas, troponin, complete blood count, and urine drug screen were within normal limits. A non-contrast head CT scan showed mild right posterior parietal swelling of unknown chronicity without evidence of intracranial hemorrhage or midline shift. A chest X-ray revealed clear lungs and a normal cardiac size and silhouette. Bedside chest, abdomen, and pelvic ultrasound were negative for free fluid and cardiac ultrasound demonstrated normal global cardiac contractility without regional wall motion abnormalities. Electrocardiogram showed normal sinus rhythm without ischemic findings. During the ED evaluation the patient’s blood pressure continued to trend upward (max 267/157), prompting initiation of a nicardipine infusion for blood pressure control.

**Figure 1:**
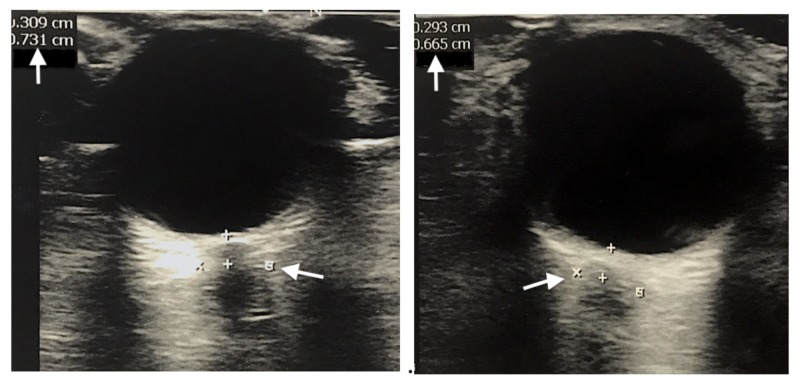
Bilateral Intra-ocular Ultrasound Optic nerve sheath diameter (ONSD) – the outer-to-outer diameter as measured 3 mm posterior to the globe (arrow): 7.31 mm in left eye (left image) and 6.65 mm in right eye (right image).

The patient was admitted and safely transferred to the medical intensive care unit (MICU) for further management. The patient remained lethargic on arrival and was noted to have intermittent non-specific shaking of the upper and lower extremities, prompting concern for seizure-like activity. Neurology was consulted and the patient was placed on electroencephalography (EEG) monitoring, which showed evidence of intermittent epileptiform sharp waves in the left temporal region, consistent with a seizure. The patient had been previously planned for an emergent lumbar puncture to assess for encephalitis. However, a concern was raised about the risks associated with performing a lumbar puncture in the setting of a seizure and increased intracranial pressure (ICP). To further evaluate for safety, a non-invasive ocular ultrasound (OUS) using a high-frequency (5-10 MHz) linear ultrasound transducer was performed with optic nerve sheath diameter (ONSD) measured 3 mm posterior to the globe to optimize optic nerve contrast. Measurements were notable for ONSD of 6.65 mm in right eye and 7.31 mm in left eye (Figure 1). Though non-diagnostic, these parameters provided influential evidence and concern for increased ICP.

**Figure 2:**
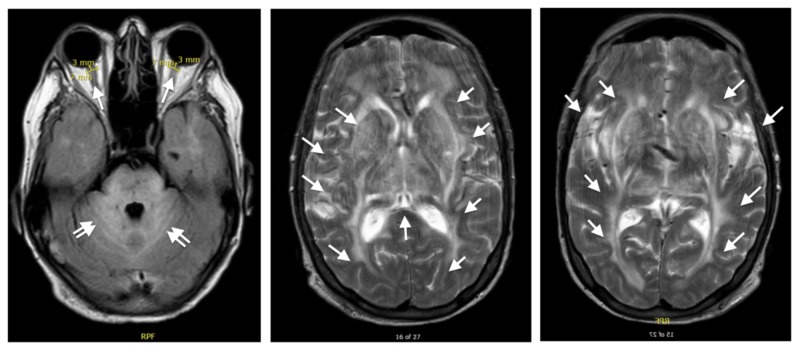
Brain MRI Fluid-attenuated inversion recovery (FLAIR) MRI showed hyperintense cerebellar white matter and brainstem changes (left image, double arrows). Measurement of optic nerve sheath diameter (ONSD) on MRI confirmed bedside ultrasonography findings of >6.5 mm ONSD bilaterally (left image, single arrows). Extensive diffuse bilateral white matter hyperintensity throughout the parietal, occipital, and temporal lobes seen on T2-weight MRI (middle and right image, arrows).

In the clinical setting of exaggerated hypertension and seizure-like activity in a patient with altered mental status, an increased concern for intracranial bleed versus cerebral edema was raised. Given the continued safety concerns for both the patient and provider of performing a lumbar puncture on a seizing, tremulous patient, the decision was made to postpone the lumbar puncture and perform an urgent MRI in order to expedite the diagnosis and treatment of the underlying pathology. The patient was given levetiracetam and midazolam for seizure control. The MRI was performed successfully, demonstrating extensive diffuse signal abnormality in the supratentorial white matter, brainstem, and cerebellar white matter (Figure 2). Measurement of ONSD on the MRI confirmed the bedside ultrasonography findings of >6.5 mm ONSD bilaterally, supportive of our initial concerns for increased ICP.

The patient’s EEG findings of seizure-like activity originating in the left anterior temporal region were consistent with the diffuse white matter change seen on MRI and supported the leading diagnosis of PRES [1]. Treatment was directed toward symptomatic relief, seizure management, and hypertensive control. The patient’s final diagnosis was PRES with deep brain involvement.

## Discussion

PRES is classically defined as a clinical disorder of reversible, subcortical vasogenic edema secondary to endothelial dysfunction [1, 2]. PRES is a misnomer as it can be both irreversible and generalized to other areas of the brain [1]. PRES is seen in both males and females of all ages [1, 2]. Common risk factors for PRES include hypertension (acute or chronic), kidney injury (acute or chronic), eclampsia, sepsis, immunosuppressive drugs, and illicit drug use (e.g., cocaine) [3].

Under normal cerebral flow autoregulation, arteriole constriction and dilation are balanced by endothelial mechanoreceptors and chemical signaling. This autoregulation is maintained when cerebral perfusion pressure (CPP) [CPP = mean arterial blood pressure (MAP) - intracranial pressure (ICP)] is maintained within the approximate range of 50-150 mmHg [4]. Outside of this homeostatic range, dysregulation occurs in the autonomic function that controls cerebral perfusion [2]. In the setting of uncontrolled hypertension or a sudden, pronounced fluctuation in CPP, a breakdown of the blood-brain vascular barrier results in hyper-perfusion of proteins and fluids into the cerebral interstitium resulting in vasogenic edema [1, 4]. If severe, this may also lead to an altered mental status or seizure activity. The posterior fossa of the brain has an increased susceptibility to dysregulation due to limited sympathetic innervation [1]. The theory of pronounced perfusion fluctuations as the underlying cause - as opposed to chronic hypertension - is supported by studies showing 15-20% of patients with PRES are normotensive or hypotensive rather than hypertensive during PRES [5].

The initial presentation of PRES is notable for the acute onset of headache, seizures, altered consciousness, and visual disturbance (cortical blindness, decreased visual acuity, hallucinations) [1, 2]. Seizures are the most common symptom, occurring in up to 90% of cases [6]. Non-convulsive status epilepticus is as common as generalized status epilepticus and should be suspected in states of prolonged altered consciousness [7]. Patients may further develop focal neurological deficits (hemiparesis or aphasia) with progression to agitation, confusion, or a comatose state [2, 3]. More than 70% of patients with PRES will present hypertensive with a mean systolic pressure ranging between 170 and 190 mmHg [3].

Labs are typically unremarkable though the acute onset kidney injury may be notable for an elevated blood urea nitrogen (BUN)-to-creatinine ratio [6]. A lumbar puncture performed in the setting of PRES to assess for meningitis or subarachnoid hemorrhage may show an elevated opening pressure but cytology would otherwise be normal [8]. Researchers have also indicated a safety concern in conducting a lumbar puncture on neurologically impaired patients in the setting of elevated ICP due to the risk of cerebral herniation [9]. A non-contrast head CT is often unremarkable, showing evidence of PRES in only approximately 50% of the cases [2]. Diffuse vasogenic edema without intracranial infarction or hemorrhage is a common finding. Intracranial hemorrhage is seen in up to 25% of cases [1]. CT-angiography will show vasoconstriction in approximately 15-30% of cases [1, 6]. EEG findings are often non-specific with a diffuse slow-wave pattern but may show sharp waves and subclinical seizures [2]. Bilateral occipital sharp waves may be present in the setting of status epilepticus [7]. MRI is considered the gold standard for PRES, with T2-weight and fluid-attenuated inversion recovery (FLAIR) MRI scans being highly sensitive for the diagnosis [10]. Abnormalities in vascular watershed areas are common in the posterior regions of both cerebral hemispheres, most commonly affecting the occipital and parietal lobes [10]. The subcortical white matter is always affected and the cortex is also often involved. The edema is typically asymmetric, but almost always bilateral [1]. Neither the pattern nor the severity of brain edema is associated with the type or severity of clinical presentation [6].

**Figure 3:**
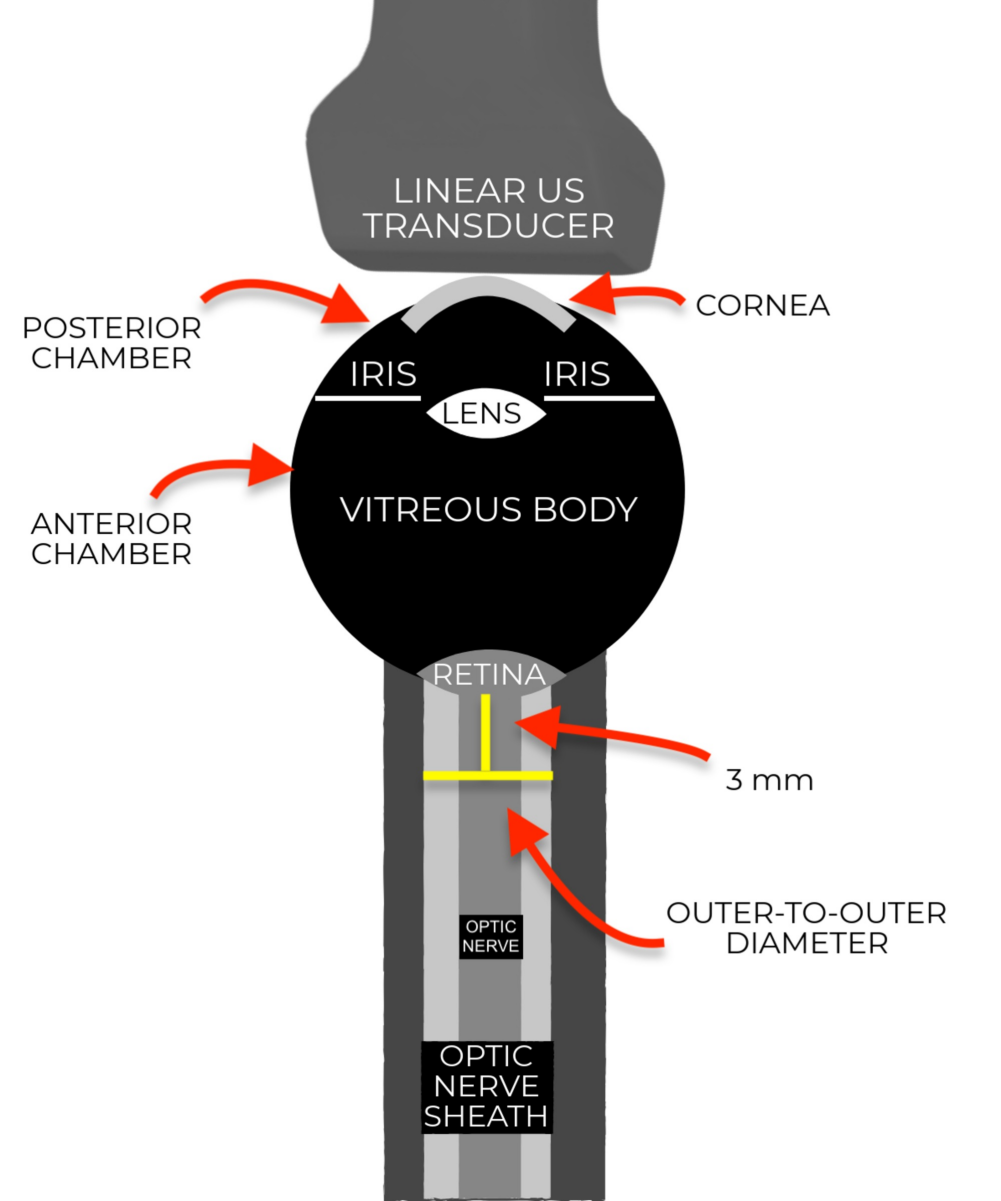
Ocular Nerve Sheath Diameter Measurement With the optic nerve centered on the screen and the lens or iris visualized to avoid false measurements, a distance of 3 mm is measured posterior to the optic disc. At 3 mm, the optic nerve sheath diameter (outer-to-outer echogenic border) is measured. (Visual created by author).

A sonographic assessment for increased ICP may aid in the early diagnosis of PRES. The utilization of ultrasound to assess critically ill patients is considered a fundamental component of emergency medicine training [11]. Predominantly used to detect retinal detachment, lens dislocation, vitreous hemorrhage, and other traumatic ocular conditions in the emergency setting [12, 13], ultrasound offers a supportive role as a non-invasive method of measuring elevated ICP due to its high intra- and inter-observer reliability [8, 14]. With the patient in supine position, a high frequency (10-15 MHz) linear transducer is placed lightly over a closed eye with copious amounts of gel to avoid excess direct pressure to the globe [13]. The optimal depth allows visualization of all ocular structures with the optic nerve centered on the screen and the lens or iris visualized to avoid false measurements. Once centered and gain optimized to avoid over-reading visual artifact, a distance of 3 mm posterior to the orbit is measured (Figure 3). At 3 mm, the ONSD is measured from outer-to-outer echogenic border [15]. To minimize intra-operator variability, three separate measurements in each eye using separate planes (six total measurements among both eyes) can be averaged to obtain an overall estimate of ONSD. A prospective study of patients with directly-measured intracranial pressure using ventriculostomy reported an ONSD greater than 5 mm was 88% sensitive and 93% specific in detecting elevated ICP (>20 cm H2O) [16]. A separate prospective study of neurocritical patients with increased ICP as confirmed via a parenchymal device reported a sensitivity of 95% and a specificity of 79% for detecting ICP when using an ONSD cutoff of 5.86 mm [17]. Although a distance of greater than 5.9 mm is often used to minimize false positives [18], providers should be aware that a significant percentage of patients with increased ICP may present below this cutoff [19]. Papilledema, defined as optic disc elevation (ODE) greater than 0.6 mm, may also be sonographically visualized in the setting of intracranial hypertension [20], though it may take hours to days to develop and is not consistently present in the acute setting.

Treatment of PRES involves the rapid reversal of offending agents and aggressive blood pressure management [2]. It is important to avoid pronounced fluctuations in blood pressure which may exacerbate PRES or lead to cerebral ischemia. Anticonvulsant therapy can be utilized to control seizures though typically not required following PRES resolution.

PRES has a favorable prognosis and is generally reversible within days to weeks of blood pressure control and symptomatic management. Persistent neurological sequelae have been reported in approximately 10 to 20% of cases and is more common in patients with uncontrolled hypertension [1].

## Conclusions

PRES is a rare but important pathology that should be considered in the differential diagnosis of any altered, seizing patient. In the case presented, sonographic findings of increased ocular pressure bilaterally were strongly suggestive of elevated ICP resulting in expedited patient care and a caution against performing an invasive lumbar puncture on a seizing patient that would have put both the patient and provider at risk of harm. Though definitive diagnosis remains an MRI, an ultrasound-guided evaluation of intra-ocular pressure in the emergency setting offers a non-invasive method of assessing for elevated ICP commonly associated with PRES.
